# Piezoelectric Sensor to Measure Soft and Hard Stiffness with High Sensitivity for Ultrasonic Transducers

**DOI:** 10.3390/s150613670

**Published:** 2015-06-11

**Authors:** Yan-Rui Li, Chih-Chung Su, Wen-Jin Lin, Shuo-Hung Chang

**Affiliations:** 1 Department of Mechanical Engineering, National Taiwan University, Taipei 106, Taiwan; E-Mails: yanray_@hotmail.com (Y.-R.L.); sudoric@gmail.com (C.-C.S.); sakungen@hotmail.com (W.-J.L.); 2 Mechanical and Systems Research Laboratories, Industrial Technology Research Institute, Hsinchu 31040, Taiwan

**Keywords:** sinus, piezoelectric sensor, ultrasonic transducer, stiffness, sensitivity

## Abstract

During dental sinus lift surgery, it is important to monitor the thickness of the remaining maxilla to avoid perforating the sinus membrane. Therefore, a sensor should be integrated into ultrasonic dental tools to prevent undesirable damage. This paper presents a piezoelectric (PZT) sensor installed in an ultrasonic transducer to measure the stiffness of high and low materials. Four design types using three PZT ring materials and a split PZT for actuator and sensor ring materials were studied. Three sensor locations were also examined. The voltage signals of the sensor and the displacement of the actuator were analyzed to distinguish the low and high stiffness. Using sensor type T_1_ made of the PZT-1 material and the front location A_1_ provided a high sensitivity of 2.47 Vm/kN. The experimental results demonstrated that our design can measure soft and hard stiffness.

## 1. Introduction

Recently, ultrasonic dental tools have been used extensively in scaling, micro-invasive implants, and other oral surgeries [1,2,3]. Ultrasonic dental tools have many benefits, such as good cutting precision, fine capability for teeth removal, simplicity of operation, and low pain effects. However, one danger during sinus lift surgery is perforation of the sinus membrane [4,5]. Although several types of ultrasonic measuring devices have been developed to diagnose dental health [6,7,8], these instruments cannot be operated simultaneously with the ultrasonic dental tool. Therefore, it is necessary to integrate a sensor into ultrasonic dental tools to avoid undesirable damage.

Past research [9] has examined the placement sensors on the bonding arm and the best sensor placement to produce signals that can be used to monitor vibration. Or and Feng [10,11] suggested recording mechanical vibrations by installing a piezoelectric (PZT) sensor in the horn to detect them. Significant changes were observed at the second harmonic of the vibration signal, and the bond shear strength could be predicted. Maruyama [12] published a technique for combining ultrasonic dental tools and dental health measurement instruments. They used a resonance frequency tracing system to detect the stiffness at the contact point during dental treatment. In common clinical cases, the operating contact force is usually below 10 N. At forces below 6 N, however, the sensitivity and resolution of the system are insufficiently high.

This work presents a method of measuring the stiffness with high sensitivity, especially in the case of soft materials. The method employs PZT materials as the sensor to measure soft and hard stiffness. By analyzing the output voltage of the sensor, the stiffness of oral tissue can be detected. We designed several types of PZT sensor using different PZT materials and tested them at different locations. Even with light contact, the sensor maintained a high stiffness sensitivity value of about 2.47 Vm/kN. The combination of actuator and sensor can be used simultaneously for dental treatment and stiffness sensing.

## 2. Design of PZT Sensor

### 2.1. Design Construction

**Figure 1:**
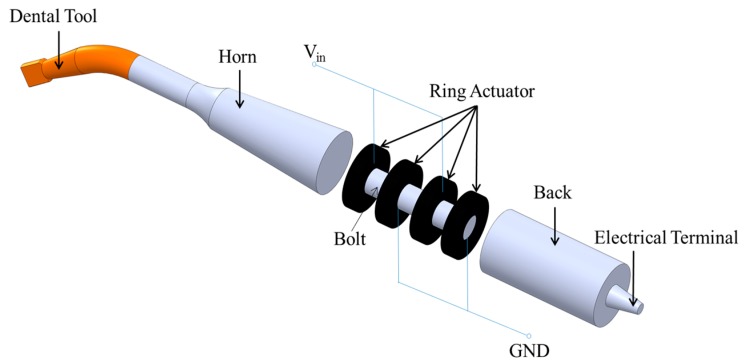
Composition of the ultrasonic dental handpiece.

A conventional ultrasonic dental handpiece tool, shown in Figure 1, consists of a dental tool and a Langevin ultrasonic transducer. The Langevin transducer includes a sandwich structure of PZT rings clamped between the horn and the back section. Longitudinal vibration is generated when the alternative voltage is applied to the PZT electrode, and the amplitude is larger at the resonant frequency. Both of the half-wavelength metal parts are configured as the displacement amplifier and are composed of four PZT rings.

**Figure 2:**
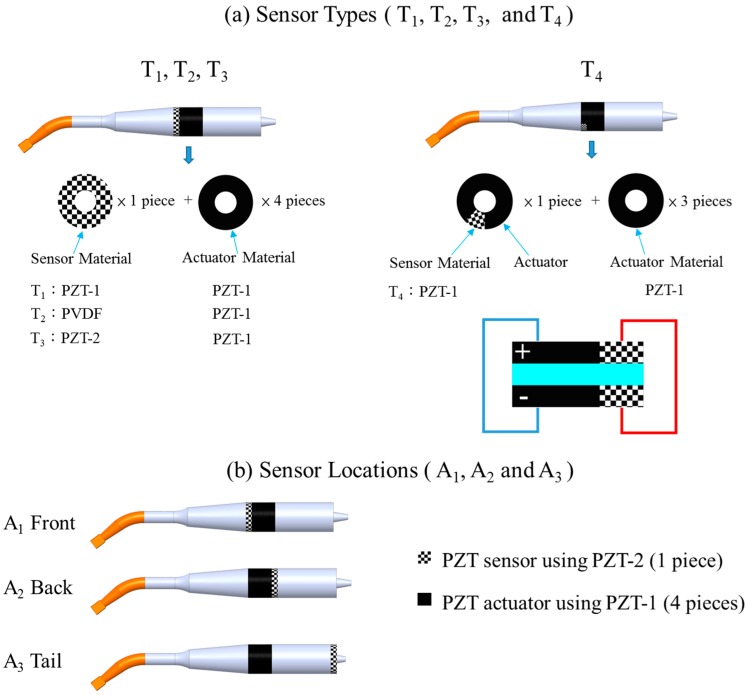
Illustration of (**a**) sensor types and (**b**) sensor locations.

A PZT ring was placed inside the transducer as a sensor, as shown in Figure 2, so that deformation of the PZT would lead to a voltage being detected. The voltage signals, having amplitudes in the ultrasonic range, and the displacement of vibration could thus be analyzed to distinguish soft and hard stiffness and thus distinguish materials of various degrees of stiffness. In this work, a PZT ring (Fuji-C213) was used as the actuator. The properties are listed in Table 1. The outer diameter, inner diameter, and thickness of the PZT ring are 10 mm, 5 mm and 2 mm, respectively.

**Table 1:** Material properties of Fuji C-213 piezoelectric (PZT) actuator.

PZT Charge Constant	PZT Voltage Constant	Dielectric Constant	Electromechanical Coupling Factor	Mechanical Quality Factor
d_33_ (pC/N)	g_33_ (×10^−3^ vm/N)	ε_33_/ε_0_	K_p_ (×10^−2^)	Q_M_
310	23.4	1470	58	2500

According to clinical findings, the ultrasonic dental handpiece is usually operated at 30 kHz, which is outside the range of human hearing, 20 to 20,000 Hz. The location and type of a sensor strongly influence the sensitivity and the displacement. These two factors will be discussed below.

### 2.2. Design of Sensor Types

As shown in Figure 2a, three different PZT materials, types T_1_, T_2_, T_3_, were studied. For type T_4_, the PZT ring was divided into an actuator and a sensor. The parameters of the sensor are listed in Table 2. The material of PZT-1 (Fuji C-213) was same as that of the PZT actuator. The PZT constant of PZT-2 (Fuji C-203) was higher than that of PZT-1, and the Young’s Modulus was similar in both. We also considered polyvinylidene fluoride (PVDF) as a material, owing to its extremely strong piezoelectricity and low density, which might make it useful for PZT sensor applications. In addition, a PZT ring was divided: 12.5% was used as a sensor, and the rest was used as an actuator. The ratio of 12.5% was chosen for two reasons. The first reason for using a split PZT as a sensor was the energy dissipation. We believed that the displacement would increase as the area ratio of the PZT sensor to the PZT actuator decreased. The second reason was that a diamond pen was used to manually cut the area we needed. The outside diameter of the PZT ring is 10 mm, and the inner diameter is 5 mm. The tip diameter of the diamond pen is approximately 1 mm. Therefore, it would be difficult to guarantee the cutting quality of a PZT ring if smaller ratios, such as 1:9 or 1:10, were used.

**Table 2:** Properties of PZT sensor materials.

Type	Parameter	Young’s Modulus	PZT Voltage Constant	Density	Thickness	Mechanical Quality Factor	Design
		Y_33_ (GPa)	g_33_ (×10^−3^ vm/N)	ρ (×10^−3^ kg/m^3^)	t (mm)	Q_M_	
T_1_	PZT-1	66	23.4	7.80	1.0	2500	Ring sensor
T_2_	PVDF	1–3	150.0	1.77	0.1	11	Ring sensor
T_3_	PZT-2	60	25.6	7.70	1.0	2000	Ring sensor
T_4_	PZT-1	66	23.4	7.80	1.0	2500	Split into sensor (12.5% area) and actuator (87.5% area)

### 2.3. Design of Sensor Locations

**Figure 3:**
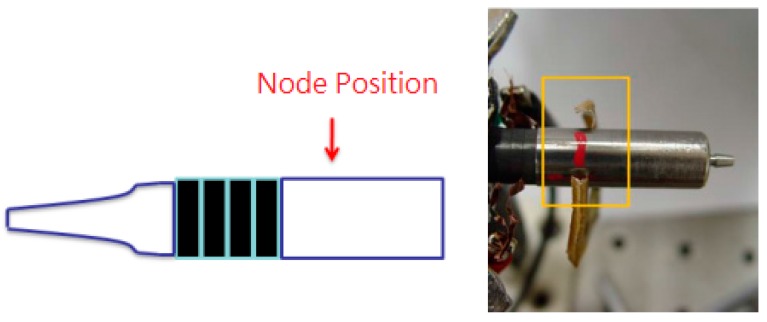
The node position of the ultrasonic dental handpiece.

Sensors at various locations generate different voltages due to the stress distribution in the transducer. The voltage of the sensor is in proportion to the force applied to the sensor. Hence, the location of a sensor will have a direct influence on its sensitivity. In this work, the sensors were placed at three different positions, *i.e.*, A_1_, A_2_ and A_3_, to find the optimal location. In the study of sensor locations, we used only PZT-1 as the actuator. As shown in Figure 3, the node was located at the position marked in red. It was reasoned that the maximum sensor voltage would be obtained with the A_2_ set for the nearest node position. Therefore, the A_1_ set for the farthest node position was the opposite of A_2_. Finally, the A_3_ set was formed by considering independent adjustment of the preload of the sensor and the actuator. As shown in Figure 2b, in position A_1_, the sensor was inserted between the ring actuator and the horn. In Position A_2_, the sensor was between the ring actuator and rear segment. In position A_3_, the sensor was glued at the tail end of the rear segment by using the instant adhesive of Loctite 403.

## 3. Experimental Measurement

This section is divided into two parts. First, the influences of the types and locations were investigated to determine the appropriate conditions for the sensor. Second, sensing capability was verified, and simply supported beams [13] with different degrees of stiffness were used as test samples.

### 3.1. Sensor Performance from Free Vibration

**Figure 4:**
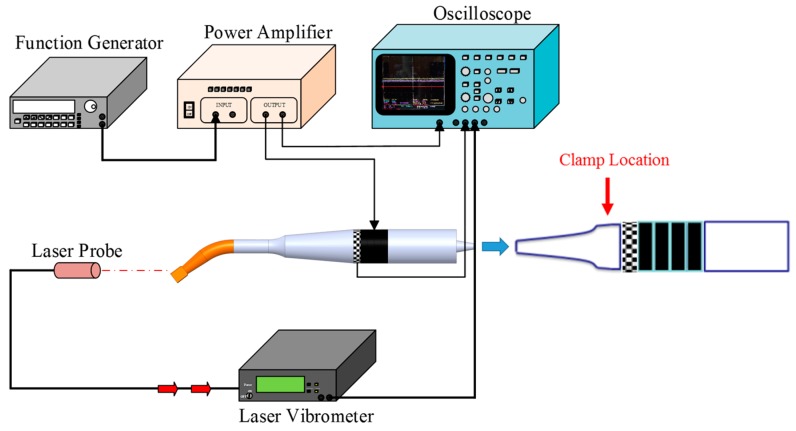
Experimental set-up for measuring the longitudinal displacement of the actuator and the sensor voltage.

**Figure 5:**
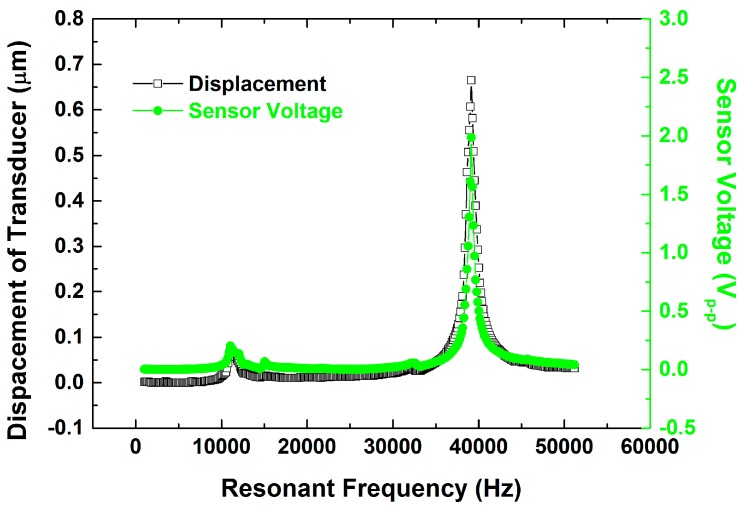
Frequency response under a driving voltage 5 V.

A free vibration test, using the set-up shown in Figure 4, was conducted to examine the efficacies of the different sensor designs. The ultrasonic transducer was clamped at a hexagonal location of the horn. The driving signal was provided by a power amplifier (NF 4005) connected to the function generator (Agilent 33120A). The longitudinal displacement of the actuator and the sensor voltage of the sensor were obtained with a laser vibrometer (Polytec OFV-3000) and an oscilloscope (Tektronix TDS 5054), respectively. The longitudinal displacement and sensor voltage were measured under a driving voltage of 5 V using a dynamic signal analyzer (HP 35665). The results are shown in Figure 5. A clear peak of the displacement and sensor voltage appeared at a resonant frequency of 40 kHz. The observed sensor voltage was consistent with the displacement. The results showed good correlation between the displacement and the sensor voltage and indicated that the sensor voltage was able to react to the displacement accurately.

### 3.2. Sensor Performance in Contact with Test Sample

Human bone tissue is composed of several layers of different materials. According to Reference [14], the bone in the upper jaw contains a high proportion of cancellous bone. The properties of polymethyl methacrylate (PMMA) are very similar to those of cancellous bone, as shown in Table 3. This work used silicon layers of thicknesses ranging from 1 mm to 5 mm to simulate the sinus membrane and PMMA to simulate human bone.

**Table 3:** Properties of cancellous bone and polymethyl methacrylate (PMMA).

Material	Young’s Modulus (MPa)	Poission’s Ratio	Density (×10^−3^ kg/m^3^)
Cancellous Bone	345	0.31	1.0
PMMA	303	0.32	1.15–1.19

**Figure 6:**
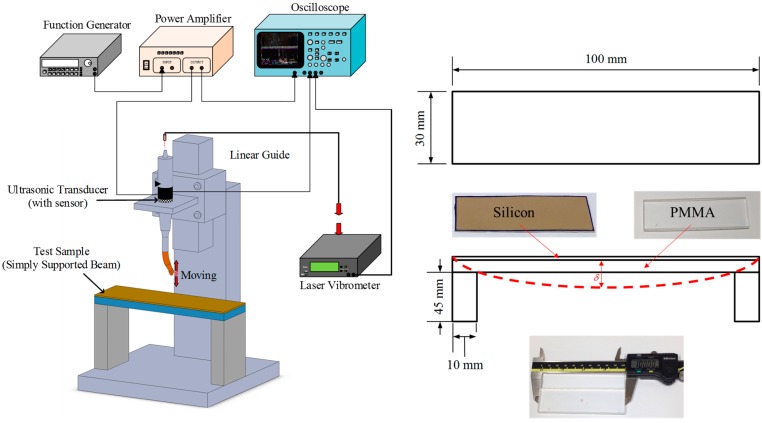
Experimental set-up for investigate sensor performance during contact with test sample.

In the experiment, simply-supported beams with different equivalent stiffness were used as test samples. The experimental apparatus of the stiffness measurement is illustrated in Figure 6. Since the tip motion at the horn was in contact with the test samples, the longitudinal displacement could be measured from the movement of the rear of the tool. The ultrasonic transducer was fixed on a slider of a linear guide and contacted the center of the test sample. During dental surgery, the conventional contact force is below 10 N. A low contact force of 3 N between the transducer and test sample was controlled by the weight of the slider and the transducer.

## 4. Results and Discussion

### 4.1. Sensor Performance in Free Vibration

**Figure 7:**
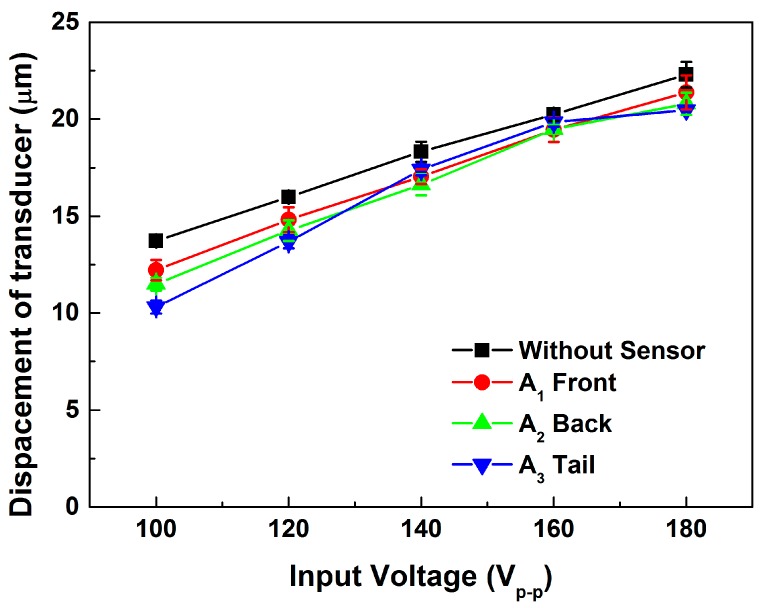
Longitudinal displacements with different sensor locations.

**Figure 8:**
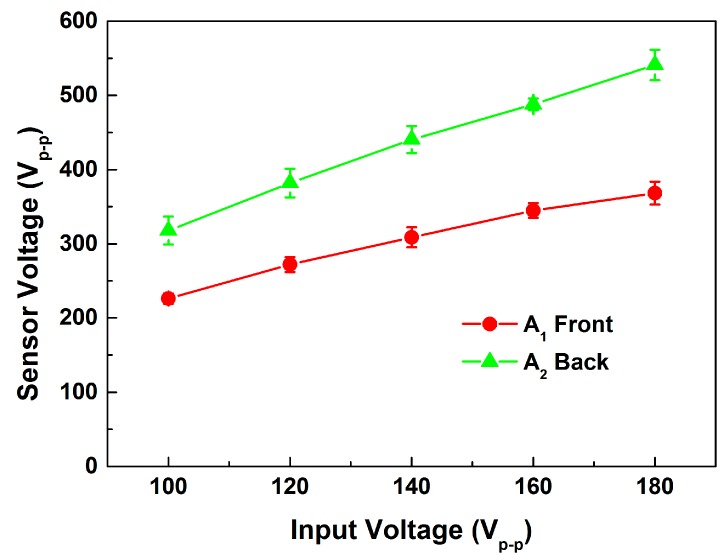
Sensor voltages of sensor locations A_1_ and A_2_.

Figure 7 shows the longitudinal displacement results for different sensor locations. In all locations, the longitudinal displacement was lower than the result for the uninstalled sensor. It can be inferred that energy was consumed when the sensors were inserted into the transducer, and some energy was converted into the sensor signal, a reasonable expectation. The results also show that the signal of the sensor location A_3_ was distorted at high input voltage; it was found that the glue become unstable under high temperature approximately 130 °C which exceeding the recommend temperature of operation. Moreover, set A_1_ produced the maximum longitudinal displacement and a lower sensor voltage than did set A_2_. According to Figure 8, the sensor voltage of A_2_ was much higher than that of A_1_. It can be inferred that the sensor voltage was higher because the sensor of set A_2_ was located close to the node of the transducer. In this work, a large displacement and sufficient voltage were needed for the device to work. Thus, the A_1_ type was used for the experimental sets T.

**Figure 9:**
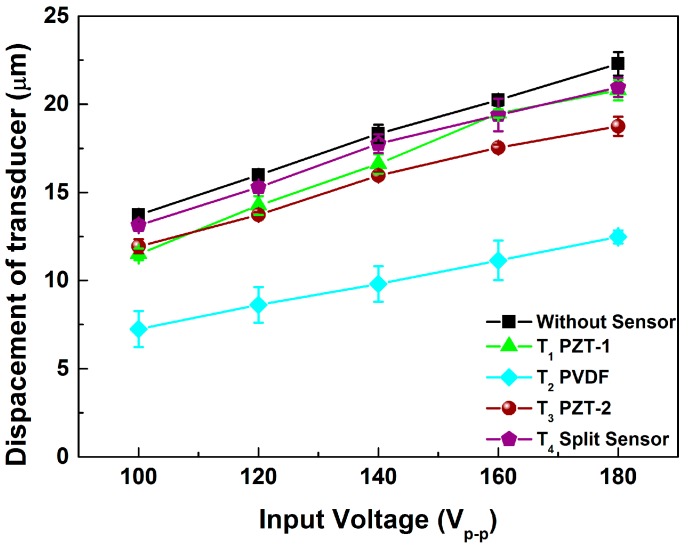
Longitudinal displacements of various sensor types.

As shown in Figure 9, experiments were performed to examine the influence of the type of sensor. The poor longitudinal displacement of sensor type T_2_ was quite noticeable and indicated that the soft PVDF material obviously lowered the transducer displacement, even though the PVDF was very thin. In addition, the displacement of T_1_ was higher than that of T_3_. About the comparison of properties of T_1_ and T_3_, the value of each item was similar, except the Mechanical Quality Factor (Q_M_). The value represents the magnitude of energy dissipation. This also indicated that a low Q_M_ corresponds to a high mechanical loss.

### 4.2. Sensor Performance in Contact Test Sample

**Figure 10:**
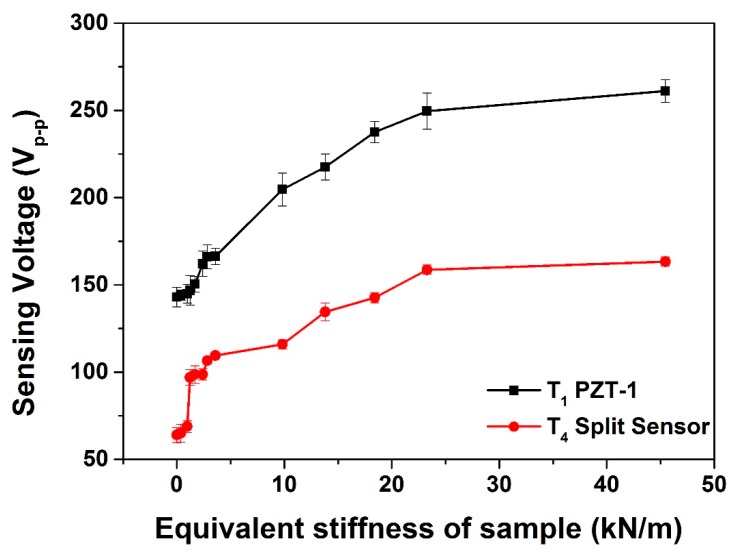
Sensor voltages at various equivalent stiffness.

Figure 10 shows the sensor voltages for the different degrees of equivalent stiffness for sensor types T_1_ and T_4_. Both designs were able to estimate the stiffness of the sample under a low contact load of 3 N. The sensor design of T_1_ showed high stiffness sensitivity of 2.47 Vm/kN, and T_4_ had a stiffness sensitivity of 1.55 Vm/kN in the stiffness range of 0 to 25 kN/m. Noticed that there is a sudden jump at 2 kN/m for type T_4_. The phenomenon was believed in the cause of the assembly-gap. For T_4_ type, a 12.5% PZT ring was divided as a sensor by a manual cutting method with the diamond pen. The method might result in the measurement error during the measurement of the low equivalent stiffness due to the slight irregularities of the sensor edge.

## 5. Conclusions

This investigation presents a new PZT sensor for integration into an ultrasonic dental tool. The sensor can distinguish hard and soft materials, even under a low contact load of 3 N. The stiffness of different test samples was detected by analyzing the voltage from the sensor. The experimental data showed that the location and the type of the sensor affected the sensitivity of the sensor and the displacement of the transducer. Measurements indicated that both type T_1_ and T_4_ were capable of distinguishing materials of different stiffness. The difference in the design of type T_4_ should be noted. This design allows a sensor voltage to be obtained without the use of an independent PZT ring as a sensor. The signal produced by this design is also sufficient for analysis, with good sensitivity of 1.55 Vm/kN. This study also determined the optimal sensor location to be A_1_, where the PZT sensor can be placed for maximum displacement of the transducer.
